# Challenges and Limitations of Targeting the Keap1-Nrf2 Pathway for Neurotherapeutics: Bach1 De-Repression to the Rescue

**DOI:** 10.3389/fnagi.2021.673205

**Published:** 2021-04-08

**Authors:** Dmitry M. Hushpulian, Navneet Ammal Kaidery, Manuj Ahuja, Andrey A. Poloznikov, Sudarshana M. Sharma, Irina G. Gazaryan, Bobby Thomas

**Affiliations:** ^1^P. A. Hertsen Moscow Oncology Research Center, Branch of the National Medical Research Radiological Center, Ministry of Health of the Russian Federation, Moscow, Russia; ^2^Faculty of Biology and Biotechnologies, Higher School of Economics, Moscow, Russia; ^3^Darby Children’s Research Institute, Medical University of South Carolina, Charleston, SC, United States; ^4^Department of Pediatrics, Medical University of South Carolina, Charleston, SC, United States; ^5^Department of Pharmaceutical Sciences, University at Buffalo, Buffalo, NY, United States; ^6^Hollings Cancer Center, Department of Biochemistry and Molecular Biology, Medical University of South Carolina, Charleston, SC, United States; ^7^Department of Chemical Enzymology, M.V. Lomonosov Moscow State University, Moscow, Russia; ^8^Department of Chemistry and Physical Sciences, Dyson College of Arts and Sciences, Pace University, Pleasantville, NY, United States; ^9^Department of Neuroscience, Medical University of South Carolina, Charleston, SC, United States; ^10^Department of Drug Discovery, Medical University of South Carolina, Charleston, SC, United States

**Keywords:** Nrf2, Kelch domain, displacement activator, ubiquitylation pathways, BACH1

## Abstract

The Keap1-Nrf2 signaling axis is a validated and promising target for cellular defense and survival pathways. This minireview discusses the potential off-target effects and their impact on future drug development originating from Keap1-targeting small molecules that function as displacement activators of the redox-sensitive transcription factor Nrf2. We argue that small-molecule displacement activators, similarly to electrophiles, will release both Nrf2 and other Keap1 client proteins from the ubiquitin ligase complex. This non-specificity is likely unavoidable and may result in off-target effects during Nrf2 activation by targeting Keap1. The small molecule displacement activators may also target Kelch domains in proteins other than Keap1, causing additional off-target effects unless designed to ensure specificity for the Kelch domain only in Keap1. A potentially promising and alternative therapeutic approach to overcome this non-specificity emerging from targeting Keap1 is to inhibit the Nrf2 repressor Bach1 for constitutive activation of the Nrf2 pathway and bypass the Keap1-Nrf2 complex.

## Introduction

**FIGURE 1:**
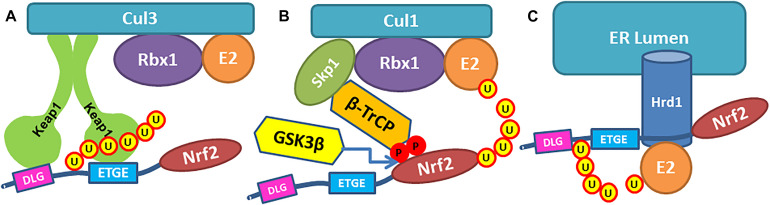
Three pathways of Nrf2 ubiquitinylation. **(A)** Nrf2 Neh2-domain is recognized by Keap1 dimer and binds Keap1 Kelch domains via ETGE and DLG binding motifs, so the lysine residues in Neh2 are ubiquitinylated by Cullin3 ligase. **(B)** β-TrCP in Cul1 ubiquitin ligase complex binds Neh6 domain in Nrf2 protein via DSAPGS and GSK3β- phosphorylated DSGIS binding motifs, so Nrf2 is getting ubiquitinylated. **(C)** Ubiquitin ligase Hrd1 ubiquitinylates Nrf2.

With the increase in the global population, the worldwide prevalence of neurodegenerative diseases is on the rise. They are among the leading causes of disability and death worldwide and will continue to grow in the coming decades due to increased life expectancy. Despite a considerable amount of basic and clinical research, most strategies to manage neurodegenerative diseases are palliative. A large body of evidence suggests that these diseases are multifactorial caused by genetic, environmental, and endogenous factors related to aging. An emerging target for neurodegenerative diseases that could modulate multiple etiological pathways involves drug-induced activation of a coordinated genetic program to maintain redox equilibrium through the expression of pro-survival proteins and cytoprotective genes (Ammal Kaidery et al., 2019; Brandes and Gray, 2020). A key transcription factor orchestrating this process is nuclear factor erythroid 2-related factor 2 (Nrf2), a member of the cap’n’collar family of basic leucine zipper transcription factors. By binding the antioxidant response elements (ARE) in promoter regions, Nrf2 regulates the transcription of over 250 genes, which together build a multifaceted network that integrates cellular activities including drug detoxification, immunomodulation, maintenance of both redox and protein homeostasis, and energy metabolism (Ammal Kaidery et al., 2019). The breadth of this endogenous response suggests that its activation might counterbalance many of the large numbers of etiological pathways implicated in neurodegenerative diseases. In recent years, therapies based on Nrf2 activation have been proposed to benefit neurodegenerative diseases (Johnson and Johnson, 2015; Gazaryan and Thomas, 2016; Cuadrado et al., 2019; Brandes and Gray, 2020). Currently, there are three known pathways for Nrf2 stabilization and activation: (a) the constitutively operating Kelch-like ECH associated protein 1 (Keap1)-dependent pathway where Nrf2 is negatively regulated by Keap1, which promotes the ubiquitination and subsequent proteasomal degradation; and (b) two recently described stress-induced pathways, where beta-transducin repeat-containing E3 ubiquitin-protein ligase (β-TrCP) and E3 ubiquitin-protein ligase synoviolin (Hrd1) negatively regulate Nrf2 through Keap1-independent mechanisms (Figure 1; Harder et al., 2015).

## Keap1-Nrf2 Pathway

**FIGURE 2:**
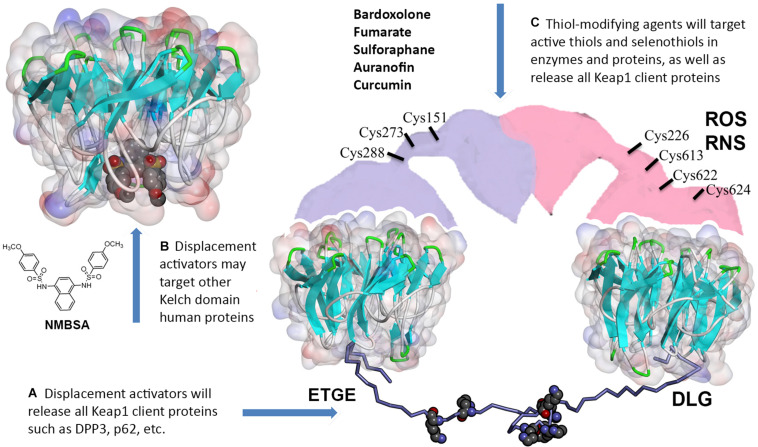
Off-target effects of Keap1 pharmacological targeting. **(A)** Thiol-modifying agents as well as displacement activators of Nrf2 (both peptides and small molecules) will release all other Keap1 client proteins. **(B)** Small molecule displacement activators may target Kelch domains in other proteins. **(C)** Thiol-modifying agents (bardoxolone and others) will inactivate redox active thiols and selenothiols in other proteins. The model for 16–85 aa Neh2 peptide (lysine residues shown with WdW radii) stretched between two Kelch domains was built using Kelch crystal structures with the corresponding Nrf2 binding motifs (4IFL.pdb and 2DYH.pdb) distanced at ca. 80 Å in accord with the Keap1 dimer conformation reported in Ogura et al. (2010). Full description of the modeling procedure is in https://www.x-mol.com/paper/1287827255333666816.

In the Keap1-dependent constitutive pathway, dimeric Keap1 (Ogura et al., 2010) recognizes the Neh2-domain in the Nrf2 protein. Keap1 binds to Nrf2 so that lysine residues in Neh2-domain are ubiquitinylated by Cullin3 (Cul3) ligase, and thus, Nrf2 is labeled for proteasomal degradation. Neh2 domain is stretched between two Kelch domains in the Keap1 dimer: Neh2 binds Kelch via DLG (weak) and ETGE (strong) binding motifs (Figures 1A, 2). As demonstrated recently^1^, the minimum recognition sequence in Neh2 domain includes 16–85 aa residues. Keap1 is a redox sensor protein (Baird and Yamamoto, 2020), and chemical modification of Keap1 Cys151 results in detachment of Cul3 ligase and stabilization of Nrf2 protein. Modification of the other redox-active cysteines in Keap1 induces conformational changes that break Keap1 dimeric structure and release Nrf2 protein (Baird and Yamamoto, 2020). A physiologically relevant Keap1 redox regulation with hydrogen peroxide is based on the formation of a disulfide bridge between Cys226/Cys613 (Fourquet et al., 2010) or any two of the Cys226/Cys613/Cys622/Cys624 residues (Figure 2), which likely results in Keap1 dimer destabilization and Nrf2 release (Suzuki et al., 2019). This constitutive pathway is characterized in detail and is commonly used to manipulate Nrf2 stability and activation.

## Nrf2 Neh6 Domain Phosphodegron

Nuclear factor erythroid 2-related factor 2 is more rapidly turned over in cells grown under homeostatic conditions than in those experiencing oxidative stress. The variable turnover of Nrf2 is accomplished through the use of at least two degrons and its redox-sensitive interaction with Keap1. McMahon et al. (2004) described a redox-insensitive degron (amino acids 329–379) in Nrf2’s Neh6- domain in 2004. They showed that if Nrf2’s half-life in Keap1 expressing cells was 10 min, in Keap1 null cells upon oxidative stress, Nrf2 was still unstable with a half-life of 40 min. The serine/threonine kinase, glycogen synthase kinase 3β (GSK3β), phosphorylates a group of serine residues in the Neh6-domain of mouse Nrf2 and promotes its degradation in a Keap1-independent manner (Rada et al., 2011; Figure 1B). There are two distinct motifs within Neh6-domain, namely DSAPGS and DSGIS, recognized by β-transducin repeat-containing protein (β-TrCP) in β-TrCP-Skp1-Cul1-Rbx1 E3/Roc1 ubiquitin ligase complex (Figure 1B). A phosphorylated DSGIS motif binds more tightly on β-TrCP than its non-phosphorylated version (Chowdhry et al., 2013). Thus, activation of GSK3β generates a phosphodegron resulting in destabilization of Nrf2. Consequently, GSK3β inhibition may result in Nrf2 stabilization and activation (in the unlikely scenario where there is no functional Keap1). Some of the reported GSK3β-inhibitors have strong pro-oxidant motifs in their structure and may likely function as direct stabilizers of Nrf2 working via the Keap1 pathway. For example, our unpublished data shows that GSK3β-inhibitor TDZD8 activates Neh2-luc reporter, which screens for direct Nrf2 stabilizers working via Keap1 (Smirnova et al., 2011). The contribution of GSK3β inhibition toward Nrf2 activation in the presence of functional Keap1 will be insignificant unless Keap1 is targeted simultaneously. This scenario also exists with TDZD8, and therefore it is not surprising that this GSK3β-inhibitor is protective in pretreatment paradigms in renal ischemia/reperfusion (Hu et al., 2016; Shen et al., 2017) or prolonged post-treatment after transplantation (Deng et al., 2020). Furthermore, GSK3β is implicated in numerous pathways, and its inhibition will unlikely be specific for Nrf2 activation. Moreover, again, in the presence of functionally active Keap1, merely inhibiting GSK3β will have no significant impact on Nrf2 activation.

## Endoplasmic Reticulum Stress-Induced Activation of Nrf2 Pathway

A more recently described pathway leading to Nrf2 destabilization is based on endoplasmic reticulum (ER)-stress-induced 3-hydroxy-3-ethylglutaryl-CoA reductase degradation protein 1 (Hrd1) (Wu et al., 2014; Figure 1C). Ubiquitin ligase Hrd1 is the focus of prevailing research since it is the critical enzyme in the complex executing retro-translocation and poly-ubiquitinylation of misfolded luminal ER proteins (Wu X. et al., 2020). However, the Hrd1-Nrf2 link is very poorly studied. The recent work claiming octyl itaconate as Hrd1 inhibitor and, as such, an Nrf2 activator (Sun et al., 2019), completely ignored the fact that octyl itaconate is a classic Michael acceptor capable of alkylating Keap1 thiols, and Cys151 in particular as shown by Liu et al. (2018). Thus, the constitutive pathway is the best-studied and is the focus of numerous efforts for the therapeutic development of Nrf2 activators. The two stress-induced pathways, and the Hrd1-Nrf2 pathway, in particular, are far from being mechanistically well-understood. They are complementary to the constitutive pathway under the conditions of acute oxidative stress. Therefore, they have to be targeted together with the constitutive pathway to reach the full benefits of Nrf2 activation.

## Nrf2 Stabilization via Targeting Keap1

Two principally different modes of Nrf2 stabilization in the constitutive pathway can be achieved: (a) irreversible, via Keap1 thiols modification followed by either dimer destabilization or Cul3 ubiquitin ligase detachment from Keap1, and (b) reversible, via competitive displacement of Nrf2 from the complex with Keap1 using Nrf2 peptides or small molecules specifically targeting Keap1 Kelch domain (Figure 2). The standard view is that the latter approach will be more beneficial because non-specific alkylation or oxidation of reactive thiols in proteins other than Keap1 can be avoided. However, one can expect off-target effects even from Nrf2 competitive displacement from Keap1, since (a) Keap1 has numerous client proteins, and (b) there are dozens of human BTB-Kelch proteins with Kelch domain structures very similar to the one in Keap1.

### Identification of Keap1 Client Proteins

The strong binding peptide motif for the Keap1 Kelch domain has the sequence of ETGE or ESGE, and mutations in these sequences disrupts Nrf2 interaction with the Keap1 Kelch-domain and results in Nrf2 activation. A comprehensive proteomics study of Keap1 interaction network (Hast et al., 2013) identified all ETGE- or ESGE-containing proteins and determined whether they functionally control Nrf2. Despite the fact that 42 Keap1-interacting proteins have been reliably identified, only 17 of those contained an ETGE, ESGE, or both motifs. The current list of verified Keap1 interacting proteins (Hast et al., 2013; Kopacz et al., 2020; Zhang et al., 2020) are shown in Table 1.

**TABLE 1:** Verified Keap1 client proteins (in addition to Nrf2).

Gene ID/*Binding motif*	Protein biological function	Role of its interaction with Keap1
AMER1 or WTX/***ETGE***	*APC membrane recruitment protein 1* forms a protein complex with protein phosphatase regulatory subunit 46 (PPP1R46 or APC), which in turn binds β-catenin into a complex with axin1, β-transducin repeat-containing protein 2 (β-TrCP2) and GSK3β, to execute ubiquitination of phosphorylated β-catenin	Role of Keap1-WTX interaction unknown
FAM129B or MINERVA/***ETGE***	Possibly regulation of Wnt/β-catenin signaling (Conrad et al., 2013)	Unknown
PALB2/***ETGE***	*Partner And Localizer Of BRCA2*: Serves as the molecular scaffold in the formation of the BRCA1-PALB2-BRCA2 complex which is essential for homologous recombination (Wu S. et al., 2020)	Keap1-mediated ubiquitination of PALB2 inhibit its function
DPP3/***ETGE***	*Dipeptidyl Peptidase 3* is a member of the M49 family of metallopeptidases, has a single zinc ion and cleaves Xaa-Pro dipeptides from the N-termini of proteins such as angiotensin, Leu-enkephalin, and Met-enkephalin (Shimamori et al., 1988; Abramic and Vitale, 1992)	DPP3-KEAP1 interaction stabilizes KEAP1 and releases Nrf2 (Lu et al., 2017)
FAM117B/***ETGE***	*Amyotrophic lateral sclerosis 2 chromosomal region candidate gene 13 protein*. Protein functions unknown	Unknown
MAD2L1/***ETGE***	*Mitotic arrest deficient-like 1* is a component of the mitotic spindle assembly checkpoint that prevents the onset of anaphase until all chromosomes are properly aligned at the metaphase plate	Unknown
MCM3/***ETGE***	*Mini-chromosome maintenance protein 3* is a subunit of the MCM2-7 complex (MCM complex) which is the putative replicative helicase essential for “once per cell cycle” DNA replication initiation and elongation in eukaryotic cells (Tamberg et al., 2018)	Keap1-mediated ubiquitination possibly inhibits MCM3 function or interferes with the complex formation
NFE2L1 (Nrf1)/***ETGE and DLG***	*Nrf1* is important for differentiation, controls the expression of proteasome, antioxidant, and metabolic genes, and regulates inflammation. Nrf1 is the most complex member of the family in terms of structure and regulation (Kim et al., 2016)	Interaction with Keap1 makes Nrf1 protein more stable (Tian et al., 2018)
IKBKB/***ETGE***	*Inhibitor of nuclear factor kappa-B kinase subunit beta* phosphorylates inhibitors of NF-κB to trigger their polyubiquitination and subsequent degradation to free and activate NF-κB. IKBKB phosphorylates other players of the pathway: NEMO/IKBKG, NF-κB subunits RELA and NFKB1, IKK-related kinases TBK1, and IKBKE (Salmeron et al., 2001; Yoboua et al., 2010)	Keap1-mediated ubiquitination results in degradation
TSC22D4/***ETGE***	*TSC22 domain family protein 4* is a transcription factor supposedly serving as a checkpoint in systemic glucose metabolism (Ekim Ustunel et al., 2016)	Binds only full-length Keap1, strongly activates Nrf2-mediated transcription
WDR1/***ETGE***	The WDR1 gene encodes *actin-interacting protein-1* (AIP1), which regulates cofilin-mediated actin depolymerization and disassembly	Binds only full-length Keap1. Role unknown
SLK/***ETGE***	*STE20-like serine/threonine-protein kinase* mediates apoptosis and actin stress fiber dissolution	SLK activates Nrf2, ETGE is dispensable
PGAM5/***ESGE***	PGAM5 is a mitochondrial protein phosphatase whose genetic ablation in mice results in mitochondria-related disorders, including neurodegeneration. Functions of PGAM5 include regulation of mitophagy, cell death, metabolism and aging (Ruiz et al., 2019; Cheng et al., 2021)	Keap1-mediated ubiquitination results in PGAM5 proteasomal degradation
SQSTM1/***STGE***	*Sequestosome-1 or ubiquitin-binding protein p62* is an autophagosome cargo protein that targets other proteins that bind to it for selective autophagy (Katsuragi et al., 2015)	Phosphorylated p62 is recognized by Keap1, p62 ubiquitination results in autophagic degradation of both cargo and Keap1
PTMA/***ENGE***	*Alpha-Prothymosin* functions in cell proliferation and differentiation, chromatin remodeling, and has antiapoptotic activity through inhibition of apoptosome formation	Mediated the import of Keap1 into the nucleus to inhibit Nrf2 activity (Khan et al., 2013)

As seen from Table 1, which lists only verified Keap1 interacting proteins via ETGE-like motifs, in many cases, the role played by such interaction is unclear. Moreover, very often, there is no ubiquitination of client proteins. Most intriguing is the recent work on Nrf1 binding to Keap1, which suggests that stretching of a peptide via ETGE and DLG between Kelch domains is not sufficient for ubiquitination and that there are specific requirements for the sequences inside the stretched peptide (Tian et al., 2018). In terms of the possibility of numerous side-effects of Keap1 targeting with either activator, the biggest concern is with the inhibitor of nuclear factor kappa-B kinase subunit beta (IKBKB), which in addition to phosphorylation of NF-κB pathway players (see in Table 1), also phosphorylates FOXO3, NCOA3, BCL10, IRS1, RIPK1, IRF5, to name just a few.

The list of Keap1 interactors covers a panoply of cellular processes, from DNA replication and licensing to cytoskeletal dynamics, transcription, and apoptosis. Predicting the plausible side effects is difficult given the absence of detailed information on Keap1 clients. However, based on the differences in the mechanism of Keap1 targeting by alkylating and displacement activators, some general predictions can be made. If we focus on thiol modifying Nrf2 activators targeting Keap1, they either destroy its dimeric structure or detach Keap1 from the ubiquitin ligase complex. In both cases, Keap1 will be no longer active with respect to ubiquitination of a client protein or its 2-point stretching between Kelch domains. However, this treatment should have no effect on the ability of individual Kelch domains to bind Keap1 client proteins via ETGE-like sequences. Contrary to this, high-affinity displacement activators targeting the Kelch domain will be capable of displacing all Keap1 interactors even if they are “hanging” onto one of the Kelch domains in the Keap1 dimer or are bound to the Keap1 monomer. So, one may expect more additional pathways to be triggered with displacement activators due to their tight binding to the Kelch domain in Keap1, which results in the release of all Keap1 dimer and monomer interactors. Displacement with peptides will be more selective since the affinity of Keap1 clients for Kelch is different, and only less tightly bound Keap1 client proteins will be displaced with a peptide (a typical dissociation constant for Nrf2-derived cell-permeable peptides is around 50 nM). So, peptide-targeted alkylating agents may turn out to be more specific than small-molecule displacement activators. However, there is an additional concern relevant to the small molecule displacement activator’s action, as we discuss below.

### Kelch Domain Proteins Besides Keap1

Small molecule displacement activators of Nrf2 are designed to target the Kelch domain in Keap1. However, Keap1 is only one among 42 Kelch-like gene family member (KLHLs) proteins in humans. Only a small portion of these proteins are currently being studied concerning their structure and functions. KLHLs are closely related to the pathogenesis of various human diseases, and specifically cancer. KLHL members associated with inherited forms of the human disease include KLHL3, KLHL7, KLHL9, KLHL12, and GAN (KLHL16), whereas KLHL6, KEAP1 (KLHL19), KLHL20, and ENC1 (KLHL37) are associated with cancer (Dhanoa et al., 2013). The KLHL family is conserved throughout evolution. According to KLHL family members’ phylogenetic analysis, it can be subdivided into three subgroups, with KLHL11 as the oldest member and KLHL9 as the youngest (Dhanoa et al., 2013). Increasing evidence shows that KLHLs exert essential biological functions through the ubiquitination of specific substrates (Shi et al., 2019). BTB-containing proteins are involved in various cellular functions such as control of the cytoskeletal organization, ion channel gating, transcription suppression, and protein targeting for ubiquitination. BTB-Kelch proteins form the largest subfamily of Cullin-RING E3 ligases, yet their substrate complexes are mapped and structurally characterized only for Keap1 and recently for some other adaptor proteins. A potential source of substrate diversity is possible through BTB cross-dimerization between different KLHL proteins, which could theoretically allow for differential substrate-binding depending on the spatial and temporal expression of KLHL proteins. Obviously, without running small molecule displacement activators against all other Kelch proteins structurally similar to the Keap1 Kelch domain, no one can guarantee the displacement activator is specific for Keap1.

We decided to pick available crystal structures of “6-blade propeller” Kelch domains and run docking for a well-known displacement activator N,N′-naphthalene-1,4-diylbis(4-methoxybenzenesulfonamide) or NMBSA, whose structure is shown in Figure 2. We found 15 structures of Kelch domains for nine proteins (KBTB5 – 4ASC.pdb; KLHDC2 – 6DO3, 6DO4, 6DO5; KLHL2 – 2XN4; KLHL3 -4CH9, 5NKP; KLHL7 – 3II7; KLHL12-6TTK, 6V7O; KLHL17 – 6HRL; KLHL20 – 5YQ4, 6GY5; NS1 protein – 5YY8, 6N3H); among those, 8 proteins, except NS1 protein (Zhang K. et al., 2018), were adaptor proteins for Cul3 ubiquitin ligase complexes. We took one structure per protein to perform NMBSA docking. To validate the docking procedure, we first docked NMBSA into the original crystal structure of Keap1 Kelch domain of (4IQK.pdb) to ensure that its docking position coincides with the actual position. Then the docking energies were taken as controls for comparison. This approach demonstrated reliable results when we used it previously to predict benfotiamine binding and its precursors to Keap1 Kelch [see docking protocol details in Tapias et al. (2018)]. As seen from Table 2, five out of the known eight crystal structures of Cul3 ubiquitin ligase adaptor proteins (or more than 60% of the available Kelch structures) showed reasonable docking energies, comparable to that for the original structure. Moreover, NMBSA docking into 3II7.pdb showed better energies than those for NMBSA docking into the original crystal structure 4IQK.pdb. Docking results do not prove that such complexes truly exist. However, their formation is permitted for these five Kelch domains, which belong to five other BTB-Kelch adaptor proteins for Cul3 ubiquitin ligase complexes. It is important to note that peptide sequences bound to various Kelch domains are not identical, and they are specific for a particular adaptor protein. Hence, using peptides with unique recognition sequences for each Kelch protein may be more specific than using small molecule displacement activators unless their specificity for Keap1 is experimentally demonstrated.

**TABLE 2:** Docking energies for NMBSA into Kelch adaptor proteins.

Kelch domain pdb ID	Protein	- CDocker energy (kcal⋅mol^–1^)	- CDocker interaction energy (kcal⋅mol^–1^)
4IQK	Keap1 adaptor protein	14.28	46.64
3II7	KLHL7 adaptor protein (Canning et al., 2013)	16.26	50.20
6DO5	KLHDC2 adaptor protein (Rusnac et al., 2018)	12.15	46.73
4ASC	KBTB5 adaptor protein (Canning et al., 2013)	6.50	43.80
5NKP	KLHL3	5.63	43.97
6GY5	KLHL20 adaptor protein (Chen et al., 2019)	8.56	40.13

## Counteracting Non-Specificity of Keap1 Targeting by De-Repression of Bach1

As we can see from the discussion on the molecular mechanisms of Nrf2 ubiquitination inhibition, there are principal concerns for using this approach to treat ongoing neurodegeneration. First, only one ubiquitination pathway is really well characterized at the molecular level – Keap1-mediated degradation of Nrf2 protein. Second, the existing thiol-modifying agents are potent inducers of the Nrf2-mediated transcriptional program and can be effectively used as preventive therapies. However, their use for existing and ongoing neurological conditions is problematic unless these agents are targeting Keap1 specifically, which is a big challenge. The excitement to use small molecule displacement activators to stabilize and activate Nrf2 by targeting the Kelch domain of Keap1 will likely diminish, as it becomes more and more evident that- (a) Keap1 has multiple client proteins, and they all will come to play upon Nrf2 displacement with high-affinity activators of this kind, and (b) there are dozens of Kelch-BTB protein adaptors for multiple ubiquitin ligase complexes having very similar structures of Kelch domains, and (c) working concentrations of displacement activators in the cells are high above their dissociation constant determined in fluorescent polarization Keap1 binding assay.

**FIGURE 3:**
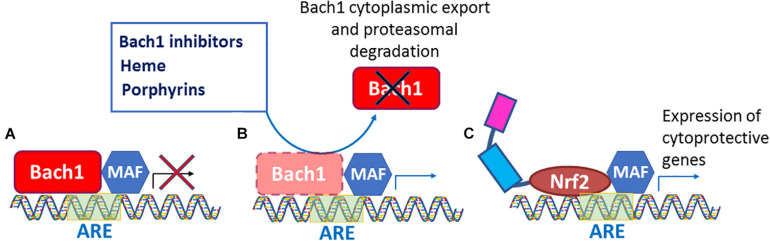
Bach1 inhibition leads to constitutive Nrf2 activation. **(A)** Bach1 binds to ARE site present on the promoter to repress Nrf2 target genes. **(B)** Bach1 inhibitors, heme, and porphyrins de-repress Bach1 resulting in its cytoplasmic export and proteasomal degradation. **(C)** The Bach1 de-repression allows Nrf2 to bind to ARE sites and leads to constitutive activation of Nrf2 pathway to overexpress cytoprotective genes.

Acute and chronic oxidative stress generates intracellular ROS and RNS, and they, as we recently learned from Suzuki et al. (2019), attack Keap1 Cys226/Cys613/Cys622/Cys624 residues (Figure 2), resulting in Nrf2 activation. As we know from numerous publications on neurodegenerative diseases, Nrf2 is stabilized under these conditions. However, it fails to bring the antioxidant genetic program to the level needed to fight against the ongoing oxidative stress. The reason for that is continuous activation of the Nrf2-driven transcriptional program that has a feedback regulation – the program triggers the expression of Nrf2 transcriptional repressors. Therefore, the higher level of Nrf2 protein is compensated by the higher level of its repressors. It is suggested that stress-induced increases in Nrf2-dependent genes decline with aging. In an interesting study, Zhou and colleagues measured basal and inducible levels of Nrf2-regulated antioxidant genes in human bronchial epithelial cells from human subjects of young adults (21–29 years) and older (60–69 years) individuals and explored factors affecting their expression. The basal expression of Nrf2-regulated genes was higher in cells from the older individuals compared with cells from the young adult. Upon exposure to an Nrf2 activator, the expression of Nrf2 genes was increased in cells from both the young adults and the older individuals; however, the induction by Nrf2 activator in older adult cells was significantly less than those observed in young adult cells. They found that the basal expression of Bach 1, the heme-responsive transcriptional repressor of Nrf2, was higher in cells from older adults than from younger adults (Zhou et al., 2018). Bach1 is an Nrf2 target gene, and as such, provide feedback mechanism counteracting Nrf2 activation. Hence, under the conditions of chronic oxidative stress, an increase in Nrf2 protein stabilization is accompanied by a simultaneous increase in the expression of Bach1. Bach1 forms heterodimers with the small Maf proteins (MafF, MafG, and MafK) to bind to MARE (Maf recognition element), thus repressing the expression of Nrf2 target genes (Oyake et al., 1996; Igarashi and Sun, 2006). As a heme sensor, Bach1 binds heme through its multiple heme regulatory motifs (Ogawa et al., 2001), thereby losing its activity as a repressor. The DNA binding activity of Bach1 dramatically decreases upon binding to heme and porphyrin-like molecules resulting in Bach1’s cytoplasmic export and its subsequent ubiquitination and degradation by the proteasome resulting in activation of Nrf2 (Figure 3). A potential alternate pathway based on pharmacological de-repression of Bach1, and thus leading to constitutive activation of the Nrf2 pathway, may result in a better therapeutic outcome when compared to Keap1 targeting. Notably, several studies have demonstrated that Bach1 inhibition/deletion is beneficial in a wide range of disorders such as spinal cord injury (Yamada et al., 2008; Kanno et al., 2009), atherosclerosis (Watari et al., 2008), ischemia/reperfusion injury (Yu et al., 2020), Huntington’s disease (Casares et al., 2020), and experimental autoimmune encephalomyelitis (So et al., 2012). The protective role of Bach1 deletion against neuronal degeneration suggests that Bach1 may represent a promising target for drug development by activating the Nrf2 pathway. Future research should focus on additional experimental studies to obtain a global view of Nrf2-mediated gene regulation via Bach1 inhibition and develop selective and safe small molecule Bach1 inhibitors as therapeutic agents. Given that Nrf2 repressor Bach1 is an Nrf2 target gene (Zhang X. et al., 2018), overexpressed in chronic oxidative stress, there is a need for a combinatorial approach involving Bach1 inhibition and Nrf2 activation as a therapeutic strategy for neurodegenerative disorders.

## Author Contributions

BT and IG conceived and wrote the manuscript. DH performed *in silico* modeling. DH, NA, MA, AP, and SS provided intellectual contribution and assisted in writing the manuscript. All authors approved the submitted version of the manuscript.

## Conflict of Interest

The authors declare that the research was conducted in the absence of any commercial or financial relationships that could be construed as a potential conflict of interest.

## Footnotes

1 https://www.x-mol.com/paper/1287827255333666816
